# Modulation of Muscle Synergies in Lower-Limb Muscles Associated With Split-Belt Locomotor Adaptation

**DOI:** 10.3389/fnhum.2022.852530

**Published:** 2022-06-30

**Authors:** Atsushi Oshima, Yasuo Nakamura, Kiyotaka Kamibayashi

**Affiliations:** ^1^Graduate School of Health and Sports Science, Doshisha University, Kyoto, Japan; ^2^Research Fellow of the Japan Society for the Promotion of Science, Tokyo, Japan; ^3^Faculty of Health and Sports Science, Doshisha University, Kyoto, Japan

**Keywords:** locomotor adaptation, split-belt treadmill, electromyography, muscle synergy, motor control

## Abstract

Humans have great locomotor adaptability to environmental demands, which has been investigated using a split-belt treadmill with belts on both the left and right sides. Thus far, neuromuscular control in split-belt locomotor adaptation has been evaluated by analyzing muscle activities at the individual muscle level. Meanwhile, in the motor control field, the muscle synergy concept has been proposed. Muscle synergies are considered the fundamental building blocks of movement and are groups of coactive muscles and time-varying activation patterns, thereby, reflecting the neurophysiological characteristics of movement. To date, it remains unclear how such muscle synergies change during the adaptation and de-adaptation processes on the split-belt treadmill. Hence, we chronologically extracted muscle synergies while walking on the split-belt treadmill and examined changes in the number, muscle weightings, and temporal activation patterns of muscle synergies. Twelve healthy young males participated, and surface electromyography (EMG) signals were recorded bilaterally from 13 lower-limb muscles. Muscle synergies were extracted by applying non-negative matrix factorization to the EMG data of each leg. We found that during split-belt walking, the number of synergies in the slow leg increased while an extra synergy appeared and disappeared in the fast leg. Additionally, the areas under the temporal activation patterns in several synergies in both legs decreased. When both belts returned to the same speed, a decrease in the number of synergies and an increase in the areas under the temporal activation patterns of several synergies were temporally shown in each leg. Subsequently, the number of synergies and the areas under the temporal activation patterns returned to those of normal walking before split-belt walking. Thus, changes in the number, muscle weightings, and temporal activation patterns of synergies were noted in the split-belt locomotor adaptation, suggesting that the adaptation and de-adaptation occurred at the muscle synergy level.

## Introduction

Humans change their walking patterns flexibly and adapt to novel and challenging walking environments. The use of a split-belt treadmill provides one way to assess adaptability (Reisman et al., 2005; Choi and Bastian, 2007). This type of treadmill can impose a novel walking condition on participants in which the speed of the belts is different on the left and right sides (i.e., split-belt condition). As an indicator that reflects an adaptation during split-belt walking, step symmetry, calculated as the difference between the step length of each leg, has been widely used in previous studies (Malone and Bastian, 2010; Bruijn et al., 2012; Yokoyama et al., 2018). In the case of healthy participants, step symmetry becomes asymmetrical in the initial phase of the split-belt condition (Bruijn et al., 2012). Interestingly, after approximately 10 min of exposure to the split-belt condition, steps become symmetrical. When the belts return to the same speed (i.e., tied-belt condition), step asymmetry appears, before becoming symmetrical again. This series of adjustments in walking patterns has been called locomotor adaptation (Reisman et al., 2010). Thus, the split-belt treadmill is useful to understand the processes of adaptation and de-adaptation simultaneously in human locomotion, and to highlight the adaptability of the human central nervous system (CNS) to changes in the environment (Torres-Oviedo et al., 2011; Helm and Reisman, 2015). Impaired split-belt locomotor adaptation has been observed in patients with neurological disorders, such as cerebellar damage (Morton and Bastian, 2006) and hemispherectomy (Choi et al., 2009), indicating that the supraspinal structures play a role in the split-belt locomotor adaptation (Hinton et al., 2020). However, how the CNS achieves split-belt locomotor adaptation remains an open question.

Electromyography (EMG)-EMG coherence analysis has been used as one of the methods for non-invasive investigation of neural control in physical movements (Halliday et al., 2003; Danna-Dos-Santos et al., 2014; Kenville et al., 2020). This analysis is a mathematical method that measures similarity in the frequency domain between a pair of EMG signals, which provides information concerning a neural drive to the motoneuron pools during physical movement (Nielsen, 2002). In particular, it has been suggested that EMG-EMG coherence in the beta band (approximately 15–35 Hz) is related to the corticospinal drive (Norton and Gorassini, 2006; Barthélemy et al., 2010). In recent years, a few studies have indicated that the EMG-EMG coherence in the beta band in an ankle dorsiflexor muscle changed when adapting walking patterns to the split-belt condition and de-adapting walking patterns to the tied-belt condition after the split-belt condition (Sato and Choi, 2019; Oshima et al., 2021).

However, previous studies using EMG-EMG coherence analysis focused on only one specific muscle. Considering that multiple muscles in the body are related to walking, knowledge obtained by focusing on one specific muscle would be insufficient to understand a neural control strategy underlying the split-belt locomotor adaptation. The muscle synergy concept has been proposed as a neural strategy to control multiple muscles relating to the generation of physical movements. This concept implies that the CNS controls a small number of modules (referred to as muscle synergies) consisting of some functionally related muscles instead of controlling muscles individually (Ivanenko et al., 2004; Dominici et al., 2011; Bizzi and Cheung, 2013). The muscle synergies are supposed to contribute to solving the degree of freedom or redundancy problem in the musculoskeletal system (Bernstein, 1967; Tresch et al., 1999). Although the origin of muscle synergies has been debated (Bizzi and Cheung, 2013; Abd et al., 2021), evidence accumulated from previous animal studies has suggested that muscle synergies are neurophysiological entities to facilitate motor control and are encoded in the spinal cord (Ting et al., 2015; Cheung and Seki, 2021). The muscle synergies in human locomotion are extracted by applying decomposition techniques, such as principal component analysis and non-negative matrix factorization (NNMF) to EMG data recorded from a large number of muscles during walking (Ivanenko et al., 2004; Yokoyama et al., 2021). A muscle synergy is represented by temporal activation pattern and muscle weighting. The temporal activation pattern is the timing of muscle activity during a gait cycle and muscle weighting is the extent of contribution to the activation pattern per individual muscle (Safavynia et al., 2011; Lacquaniti et al., 2012). Previous studies have shown that in healthy adults four or five muscle synergies can explain the variability in muscle activity patterns during normal walking (Cappellini et al., 2006; Ivanenko et al., 2006). It has also been indicated that each extracted muscle synergy has a particular function in a gait cycle (e.g., weight acceptance or propulsion during the stance phase) (Neptune et al., 2009; Lacquaniti et al., 2012). Thus far, presence of neurological disorders (Clark et al., 2010), development from neonatal to toddler stages (Dominici et al., 2011), and walking speeds (Yokoyama et al., 2016) have been reported to alter the number of muscle synergies. Further, the temporal activation patterns have been shown to be modified according to the walking speed (Hagio et al., 2015; Kibushi et al., 2018) and walking surfaces (Martino et al., 2015; Santuz et al., 2018). The muscle weightings recruited during an imposed walking task have been shown to be dependent on the training history (Sawers et al., 2015). Thus, the number, temporal activation patterns, and muscle weightings of muscle synergies may change depending on the situation during walking. Hence, studies focusing on such muscle synergies can deepen the understanding of neuromuscular control underlying the split-belt locomotor adaptation. In a recent study using cats, muscle synergies were analyzed on a split-belt treadmill. However, changes in muscle synergies associated with adaptation and de-adaptation were not investigated (Desrochers et al., 2019). MacLellan et al. (2014) have extracted muscle synergies in the initial and late phases of split-belt walking and tied-belt walking following split-belt walking in humans but did not examine changes in muscle synergies over time. Therefore, it remains unclear whether muscle synergies change gradually or quickly in the adaptation and de-adaptation processes on the split-belt treadmill.

When examining changes in muscle synergies over time on the split-belt treadmill, one of the following patterns is expected to occur, given the hypotheses proposed in previous studies (Severini et al., 2020; Abd et al., 2021):

1) changes in the number of muscle weightings and temporal activation patterns
2) no changes in the muscle weightings but changes in the temporal activation patterns
3) changes in the muscle weightings and temporal activation patterns
4) both 1 and 3

Thus, in this study, we aim to investigate changes in muscle synergies over time and test which one of the four abovementioned hypotheses is valid in the adaptation and de-adaptation processes on the split-belt treadmill. To accomplish this, we extracted muscle synergies from the lower-limb muscles chronologically during split-belt walking and tied-belt walking after split-belt walking. The present study may provide new knowledge about the neuromuscular control in the split-belt locomotor adaptation.

## Materials and Methods

### Participants

Twelve healthy young men (22.1 ± 2.9 years, 170.3 ± 5.5 cm, and 62.9 ± 4.5 kg) participated in this study. The dominant leg was the right leg in all participants. We excluded participants with neurological impairments. All participants provided written informed consent prior to participating in the experiment. The procedures in this study were approved by the Doshisha University Research Ethics Review Committee regarding Human Subject Research, and this study was performed in accordance with the Declaration of Helsinki. Participants who had experience walking on a split-belt treadmill before were not included in the present study.

### Experimental Design

**FIGURE 1:**
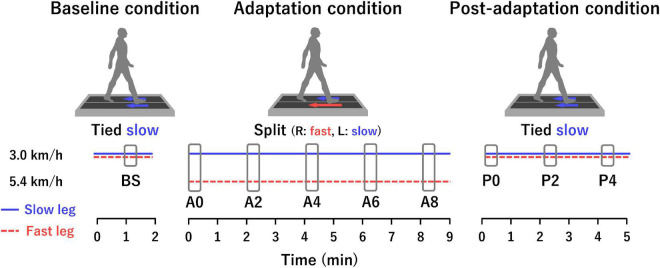
Experimental paradigm and analysis sections. Tied is a belt condition in which both belts move at the same speed. Split is a belt condition in which each belt moves at a different speed. In the adaptation condition, the right belt was fast (5.4 km/h), and the left belt was slow (3.0 km/h) for all participants. The horizontal blue solid and red dotted lines represent the slow leg and fast leg, respectively. The horizontal axis indicates time. Each rectangle in the baseline, adaptation, and post-adaptation conditions represents the analysis sections consisting of 20 gait cycles.

We applied an experimental design established by Bastian and colleagues (Reisman et al., 2005). The participants walked on a split-belt treadmill (HPT-1980D-DU, Tec Gihan Co., Ltd.) with two belts controlled separately by independent motors. The treadmill was operated in either a tied-belt condition (i.e., the two belts move at the same speed) or a split-belt condition (i.e., the two belts move at different speeds). The belts moved from the front to the back throughout the experiment. The belt speed was set at 3.0 km/h (slow) or 5.4 km/h (fast). Figure 1 illustrates the experimental paradigm. The baseline condition was the tied-belt condition at 3.0 km/h for 2 min. The adaptation condition was the split-belt condition with the left belt at 3.0 km/h and the right belt at 5.4 km/h for 9 min (belt speed ratio of 1:1.8). The leg moving faster during the adaptation condition was assigned to the dominant leg in all participants. We defined the leg on the slow and fast belts during the adaptation condition as the “slow leg” and the “fast leg,” respectively. In the post-adaptation condition, the belt condition was again the tied-belt condition at 3.0 km/h for 5 min. The belts were stopped between the baseline and adaptation conditions, but not between the adaptation and post-adaptation conditions. All changes in belt speeds (e.g., from 5.4 to 3.0 km/h between the adaptation and post-adaptation conditions) took 5 s. Participants were verbally informed of the next belt speed by an experimenter about 10 s before the actual changes in the belt speed. They were instructed to look at the centerline on a screen about 2 m ahead without looking down as much as possible while walking. All participants wore a safety harness around the upper chest to prevent falls during walking. The harness was mounted on the suspension device, but it did not support their body weight. Additionally, emergency buttons were placed within the reach of the participant and experimenter.

### Data Collection

Kinematic data were collected at 100 Hz using a motion capture system with eight cameras (OptiTrack motion capture system, NaturalPoint Inc.). Infrared reflective markers were attached bilaterally on the ankles (lateral malleolus). Three-dimensional ground reaction force (GRF) data [mediolateral (Fx), anterior-posterior (Fy), and vertical (Fz) components] were recorded at 1,000 Hz from a force plate mounted underneath each belt of the treadmill (TFH-40120-EL and TFH-40120-ER, Tec Gihan Co., Ltd.). Surface EMG electrodes (Trigno Wireless System, Delsys Inc.) were used to record EMG bilaterally from the following 13 muscles: gluteus maximus (Gmax), gluteus medius (Gmed), adductor longus (AL), tensor fasciae latae (TFL), biceps femoris (BF), semitendinosus (ST), rectus femoris (RF), vastus lateralis (VL), vastus medialis (VM), medial gastrocnemius (MG), lateral gastrocnemius (LG), soleus (SOL), and tibialis anterior (TA) muscles. The electrode locations were determined by referring to the Surface Electromyography for the Non-Invasive Assessment of Muscles (SENIAM) guidelines^1^ and confirmed using an ultrasonic device (Prosound α7, Hitachi-Aloka Medical, Ltd.). Before placing the electrodes, the skin was lightly rubbed with fine sandpaper and cleaned with alcohol swabs. The recorded EMG signals were amplified (with a 300-gain preamplifier) before further amplification (total effective gain of 909), band-pass filtered (20–450 Hz), and stored on a computer for later analyses after A/D conversion at 1,000 Hz (PowerLab 16/35, AD Instruments Inc.). The timing for recording the kinematic, GRF, and EMG data was synchronized.

### Data Analysis Section

Multiple analysis sections were set for the analyses of the spatio-temporal parameters and the muscle synergy (Figure 1). Each section consisted of 20 consecutive gait cycles. One section was set from 1 min after the start of the baseline condition (BS). In the adaptation condition, five sections were set as follows: immediately after the start of the adaptation condition (A0), 2 min after the start (A2), 4 min after the start (A4), 6 min after the start (A6), and 8 min after the start (A8). In the post-adaptation condition, three sections were set as follows: immediately after the start of the post-adaptation condition (P0), 2 min after the start (P2), and 4 min after the start (P4).

### Analysis of Spatio-Temporal Parameters

The kinematic and GRF data were low-pass filtered at 6 and 15 Hz, respectively (Reisman et al., 2005; Sato and Choi, 2019). From the Fz component of the GRF, the timings of the heel strike and toe-off of each leg were determined (threshold: 5% of the bodyweight). Based on the timings of the heel strike and toe-off, stance time, swing time, and double support time were calculated. These temporal parameters were normalized to the duration of one gait cycle.

From the kinematic data, step symmetry was calculated. Step symmetry was defined as the normalized difference between the step length of each leg following the equation:


Step symmetry

$$\;=\frac{\text{Step length of fast leg}-\text{Step length of slow leg}}{\text{Step length of fast leg}+\text{Step length of slow leg}}$$

**FIGURE 2:**
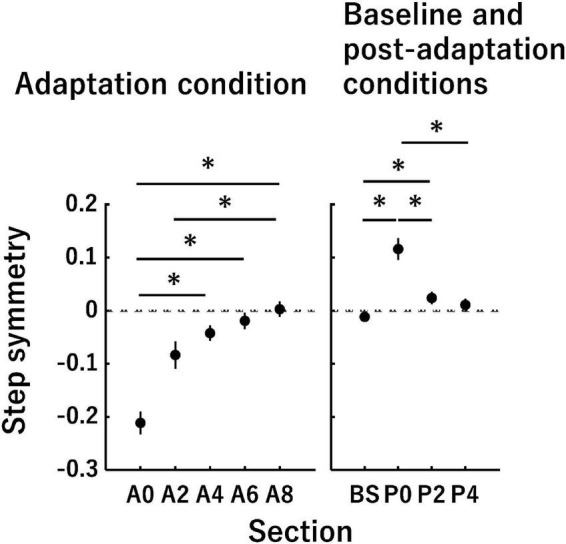
Time-series changes in the step symmetry. The vertical axis indicates the step symmetry, and the horizontal axis indicates the sections in each panel. A value of 0 in the vertical axis (dotted lines) indicates that the step lengths of the slow leg and fast leg are equal (i.e., symmetry). The panels represent time-series changes in the step symmetry in the adaptation condition (left panel) as well as the baseline and post-adaptation conditions (right panel). Error bars indicate mean ± SE. *P < 0.0051 (adaptation condition) and 0.0085 (baseline and post-adaptation conditions).

In this equation, the step length was the anterior-posterior distance between the ankle markers of each leg at the heel strike of the leading leg (Reisman et al., 2005). The step length for the slow leg was measured at the heel strike of the slow leg. A positive value of the step symmetry indicates that the step length of the fast leg was longer than that of the slow leg (i.e., asymmetry) (Figure 2). A value of 0 indicates that the step lengths of the fast leg and slow leg are equal (i.e., symmetry).

### Muscle Synergy Analysis

Muscle synergy analysis was performed in the slow leg and fast leg, respectively. Twenty consecutive gait cycles at each section were used to extract muscle synergies (Oliveira et al., 2016). The recorded EMG signals were high-pass filtered (40 Hz) with a zero-lag fourth-order Butterworth filter, demeaned, full-wave rectified, and low-pass filtered (10 Hz) with a zero-lag fourth-order Butterworth filter (Clark et al., 2010; Kibushi et al., 2018). We confirmed visually that there were no obvious artifacts in the smoothed EMG signals. The smoothed EMG data were time-interpolated to 200-time points per one gait cycle (Cappellini et al., 2016). The EMG envelopes were then ensemble-averaged (Nazifi et al., 2017; Saito et al., 2018). Thus, the EMG matrix, which consisted of 13 rows and 200 columns was generated in each leg at each section. The EMG amplitude of each muscle in each matrix was normalized to the maximum amplitude across the sections used in the statistics (see “Statistics” section: A0–A8 for the adaptation analysis, and BS and P0-P4 for the de-adaptation analysis) per participant (Yokoyama et al., 2019), indicating that the amplitude of each muscle was scaled from 0 to 1. Then, the data of each muscle in each matrix was normalized to the standard deviation of that muscle to have unit variance (Chvatal and Ting, 2012; Hagio and Kouzaki, 2014). This normalization was removed after extracting muscle synergies to rescale the data to the original scaling.

The NNMF was used to extract muscle synergies from each EMG matrix (Lee and Seung, 1999; Yokoyama et al., 2016; Boccia et al., 2018), which has previously been described as a linear decomposition technique according to the following equation:


E=W⋅C+e=Er+e

In this equation, *E* is an *m* × *t*matrix [where *m* is the number of muscles (13) and *t* is the time point (200)] that is an original EMG matrix, *W* is an *m* × *n* matrix (where *n* is the number of muscle synergies) that indicates muscle weightings, *C* is an *n* × *t* matrix that indicates temporal activation patterns, *e* is the residual error matrix, and *Er* is a reconstructed EMG matrix from the multiplication of *W* and *C*. Each vector in the extracted muscle weightings (each column of *W*) was normalized to its maximum and each vector in the extracted temporal activation patterns (each row of *C*) was scaled by the value used in the normalization of muscle weighting corresponding to the temporal activation pattern. Thus, each vector in muscle weightings was a unit vector.

**FIGURE 3:**
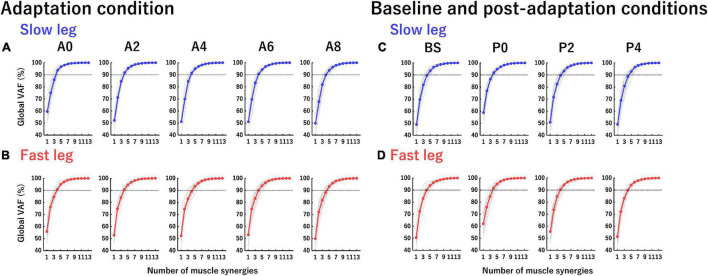
Global variability accounted for (VAF) curves in each leg at each section. The vertical axis indicates global VAF (%) and the horizontal axis indicates the number of muscle synergies. Horizontal dotted lines at 90% VAF indicate the threshold of global VAF. **(A,B)** Global VAF curves of the slow leg and fast leg at each section in the adaptation condition. **(C,D)** Global VAF curves of the slow leg and fast leg at each section in the baseline and post-adaptation conditions. Thin gray curves indicate the global VAF curve of each participant and colored curves (blue and red) indicate the averaged global VAF curves from all participants.

The extraction was performed using a possible *n* between 1 and 13 and in each *n* the extraction was iterated 100 times. To select the optimal number of muscle synergies, in each *n*, a goodness of fit between the original EMG matrix (*E*) and reconstructed EMG matrix (*Er*) was calculated using the variability accounted for (VAF). The VAF describes the extent to which the variability of the original EMG data was accounted for by the reconstructed EMG data. The value of VAF was calculated as 100 × the coefficient of determination from the uncentered Pearson correlation coefficient in the entire EMG data (global VAF) and each muscle EMG data (muscle VAF) (Hagio et al., 2015; Figure 3). The optimal number of muscle synergies was defined as the minimum number of muscle synergies required to achieve a global VAF > 90% and a muscle VAF > 75% (Barroso et al., 2014; Boccia et al., 2018; Kibushi et al., 2018). After determining the optimal number of muscle synergies in each participant, we determined the number of muscle synergies at each section as a rounded mean number of muscle synergies across participants for further analysis.

**FIGURE 4:**
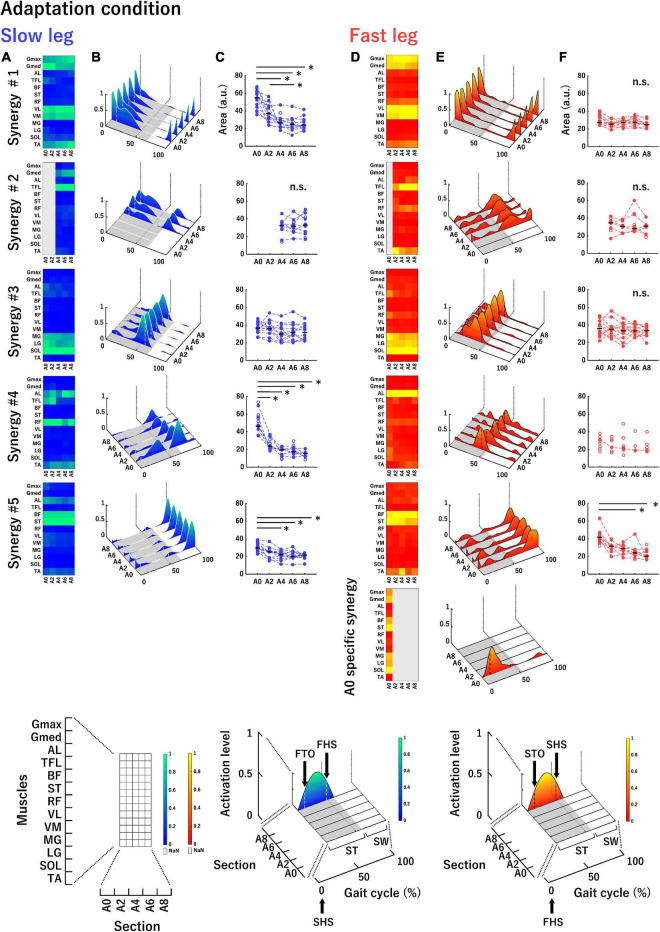
Average muscle synergies across participants in the slow leg **(A–C)** and the fast leg **(D–F)** at each section in the adaptation condition. **(A,D)** Each heatmap represents the muscle weightings of muscle synergies. The vertical axis indicates muscle names, and the horizontal axis indicates sections. Bright color (green or yellow) indicates high weighting. The parts filled with gray in the heatmap mean that a corresponding muscle synergy was not extracted. **(B,E)** Each waveform represents the temporal activation patterns of muscle synergies. The vertical axis indicates activation level and the two axes in the bottom plane indicate sections and gait cycle (%). Bright color (green or yellow) indicates high activation. The bottom part of the figure, shown with gray, represents stance phase and the vertical white or black dotted lines depicted within the temporal activation patterns represent boundaries of the double support phase. An enlarged view of each axis of each heatmap and waveform is shown in the lowest row. **(C,F)** The areas under the curves of the temporal activation patterns of muscle synergies. The vertical axis indicates the area (a.u.) and the horizontal axis indicates the sections. The area of each participant is denoted by circles. The filled circles connected by dotted lines were used in the statistical analysis. Horizontal black bars indicate the median value across participants. n.s. indicates no significant difference in the one-way repeated measures ANOVA or Friedman test. ST, stance phase; SW, swing phase; FTO, fast leg toe-off; FHS, fast leg heel strike; STO, slow leg toe-off; SHS, slow leg heel strike. *P < 0.0051.

**FIGURE 5:**
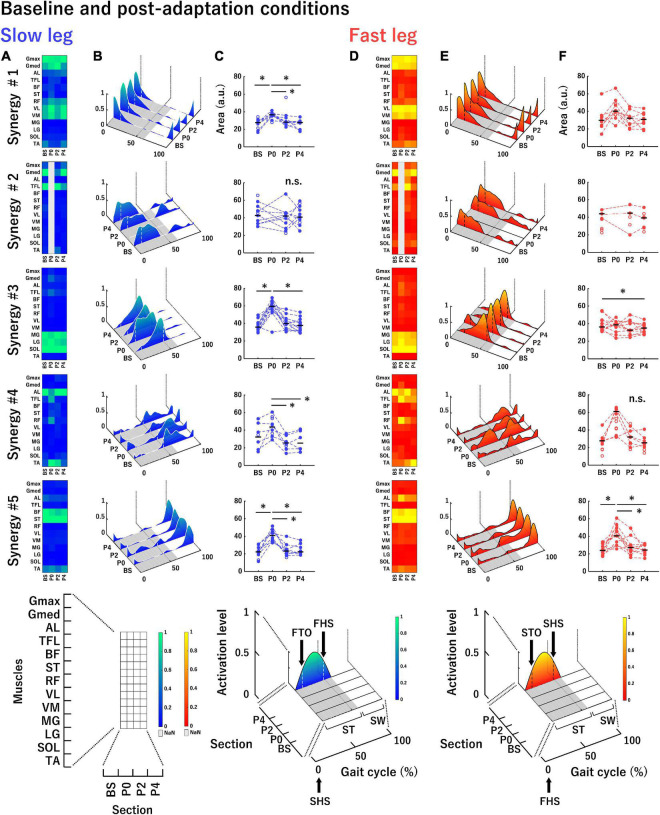
Average muscle synergies across participants in the slow leg **(A–C)** and the fast leg **(D–F)** at each section in the baseline and post-adaptation conditions. **(A,D)** Each heatmap represents the muscle weighting of muscle synergies. **(B,E)** Each waveform represents the temporal activation patterns of muscle synergies. **(C,F)** The areas under the curves of temporal activation patterns of muscle synergies. The conventions of each panel are the same as those in Figure 4. *P < 0.0085.

To sort the muscle synergies extracted by the rounded mean number of muscle synergies in all participants at each section, the cosine similarity value that was calculated as a scalar product between a pair of vectors normalized by the product of the norm of each vector was used (Oliveira et al., 2014; Singh et al., 2020). The cosine similarity values close to 0 and 1 were considered dissimilar and highly similar, respectively. In this study, when the cosine similarity value was over 0.684 (*P* < 0.01), the pair of vectors were determined to be similar. First, the cosine similarity values between each vector in the muscle weightings of an arbitrary reference participant and that of the remaining participants were calculated (Hagio et al., 2015; Kibushi et al., 2018), and an average muscle weighting set was made using similar vectors. Subsequently, the cosine similarity values were again calculated between each vector in the average muscle weighting set and that in the muscle weightings of each participant. If two vectors in one participant were categorized into one vector in the average muscle weighting set, a vector with the highest correlation was selected. The average muscle synergies, consisting of the muscle synergies finally categorized as similar muscle synergies across participants by sorting vectors of muscle weightings, were then made. Through these steps, several muscle synergies in some participants were not included in any of the average muscle synergies. We also calculated the cosine similarity value of each vector in the muscle weightings of the average muscle synergies between A0 and the remaining sections in the adaptation condition, as well as between the BS and all sections in the post-adaptation condition. The average muscle synergies that were similar across sections are shown in Figures 4, 5. The principal muscles within similar muscle synergies across sections were defined as muscles that showed weighting values ≥ 0.5 in more than half of the number of sections for statistics (see Statistics section: A0–A8 for the adaptation analysis and BS and P0–P4 for the de-adaptation analysis) (Tables 1, 2). We calculated the areas under the curves of the temporal activation patterns in participants included in the average muscle synergies at each section to investigate changes in the temporal activation patterns (Hayes et al., 2014; Sawers et al., 2015). All data processing and analysis were performed using custom software written in MATLAB R2020b (MathWorks Inc.).

**TABLE 1:** Principal muscles and main activation timing of each muscle synergy in the adaptation condition.

Adaptation condition		
	Principal muscles	Timing
Synergy #1	Gmax, Gmed, VL, VM	Initial double stance
Synergy #2	TFL	Stance ⋅ Early swing
Synergy #3	MG, LG, SOL	Late stance
Synergy #4	AL	Terminal double stance
Synergy #5	BF, ST	Late swing
Specific synergy (*F*)	Gmed, BF, ST, MG, LG, SOL	Initial double stance ⋅ Single limb support

*F, Fast leg.*

**TABLE 2:** Principal muscles and main activation timing of each muscle synergy in the baseline and post-adaptation conditions.

Baseline and post-adaptation conditions		
	Principal muscles	Timing
Synergy #1	Gmax, Gmed, VL, VM	Initial double stance ⋅ Mid stance
Synergy #2	Gmed, TFL	Initial double stance ⋅ Single limb support
Synergy #3	MG, LG, SOL	Late stance
Synergy #4	AL	Early swing
Synergy #5	BF, ST	Late swing

### Statistics

We focused on the respective changes in the muscle synergies of each leg when adapting walking patterns to the split-belt condition and de-adapting walking patterns to the tied-belt condition after the split-belt condition. Thus, statistical analysis was performed using the following combination of sections: A0–A8 for the adaptation analysis, and BS and P0–P4 for the de-adaptation analysis. Regarding the statistical analysis of the areas of the temporal activation patterns, when the number of participants who recruited a certain muscle synergy throughout all sections selected for each statistical analysis of adaptation and de-adaptation was more than six (see filled markers and dotted lines in Figures 4C,F, 5C,F), we performed the statistical analysis on those participants. The statistical analysis for step symmetry was conducted on all participants in the same combination of sections. The normal distribution of data was evaluated using the Shapiro–Wilk test. If the data at each section used in each statistical analysis were normally distributed, we performed a one-way repeated-measures analysis of variance (ANOVA). When the assumption of sphericity (Mauchly’s test) was violated, Greenhouse–Geisser adjustments were applied to adjust the degrees of freedom. When statistical significance was found using the repeated-measures ANOVA (Significance level alpha = 0.05), Sidak–correction *post hoc* comparisons [1-(1- significance level alpha of 0.05) ^ (1/number of compared pairs)] were performed to examine the differences among sections [Sidak-adjusted alpha level for the adaptation analysis = 0.0051 (10 pairs), and sidak-adjusted alpha level for the de-adaptation analysis = 0.0085 (six pairs)]. If the data were not normally distributed, a non-parametric Friedman test was conducted. When statistical significance was found using the Friedman test (Significance level alpha = 0.05), the Wilcoxon signed-rank sum test (Sidak–correction) was used to examine the differences among sections. Data are presented as mean ± standard error (SE). All statistical analyses were performed using SPSS (IBM SPSS Statistics for Windows, Version 27.0, IBM Corp.).

## Results

### Step Symmetry

Figure 2 shows the time-series changes in the step symmetry. Although the step symmetry exhibited a negative value in the initial phase of the adaptation condition, it gradually approached 0 by the late phase of the adaptation condition. In the initial phase of the post-adaptation condition, the step symmetry became a positive value despite the tied-belt condition being identical to the baseline condition. This value of step asymmetry reduced gradually toward the baseline value. In the adaptation condition, there was a significant main effect of section [Chi^2^ (df = 4) = 40.00, *P* < 0.05]. The *post-hoc* tests showed significant differences in the following pairs of sections: A0–A4, A0–A6, A0–A8, and A2–A8 (all *P* < 0.0051). In the baseline and post-adaptation conditions, there was a significant main effect of section [*F*_(1.30, 14.36)_ = 36.72, *P* < 0.05]. The step symmetry at P0 was significantly different compared with that at BS, P2, and P4 (all *P* < 0.0085), and there was also a significant difference between BS and P2 (*P* < 0.0085).

### Number of Extracted Muscle Synergies

Figure 3 shows the global VAF curves in each leg at each section in the adaptation condition (Figures 3A,B) and the baseline and post-adaptation conditions (Figures 3C,D). The mean number of muscle synergies in the slow leg at each section was as follows: 4.17 ± 0.21 (A0), 4.42 ± 0.15 (A2), 4.50 ± 0.15 (A4), 4.58 ± 0.15 (A6), 4.75 ± 0.18 (A8), 4.75 ± 0.13 (BS), 4.17 ± 0.17 (P0), 4.92 ± 0.19 (P2), and 4.92 ± 0.23 (P4). Thus, we determined the number of muscle synergies at A0, A2, and P0 to be four and the other sections to be five. The mean number of muscle synergies in the fast leg at each section was as follows: 4.75 ± 0.13 (A0), 4.75 ± 0.18 (A2), 4.67 ± 0.22 (A4), 4.83 ± 0.27 (A6), 4.92 ± 0.23 (A8), 4.75 ± 0.18 (BS), 4.42 ± 0.19 (P0), 4.92 ± 0.23 (P2), and 5.17 ± 0.17 (P4). Therefore, the number of muscle synergies was five in all sections, except for four muscle synergies at P0.

### Extracted Muscle Synergies in Adaptation Condition

Figure 4 shows the average muscle weightings (heatmaps, Figures 4A,D), average temporal activation patterns (waveforms, Figures 4B,E), and areas under the curves of temporal activation patterns (scatter plots, Figures 4C,F) in each leg at each section in the adaptation condition. In Figures 4B,E, the average stance time at each section is indicated with the gray area at the bottom surface of the figure. In addition, the average time of double stance and single limb support in each section is identified by the timing of the heel strike or toe-off in the contralateral leg, indicated by the vertical dotted lines in the temporal activity patterns. The muscle synergies were aligned based on the timing of the activation during a gait cycle from stance to swing. The principal muscles and main activation timings in each muscle synergy are summarized in Table 1. Synergy #1 mainly represented the activation of the Gmax, Gmed, VL, and VM. These muscles were mainly active during the initial double stance phase. They were also active during single limb support phase in the slow leg at A0 and A2. Synergy #2 mainly represented the activation of the TFL, which was active during the stance phase and early swing phase. This synergy was not recruited in either leg at A0 and in the slow leg at A2. Synergy #3 represented the activation of the MG, LG, and SOL, which were mainly observed during the late stance phase. Synergy #4 mainly represented the activation of the AL in both legs and the RF in the slow leg. The muscles in this synergy were active during the terminal double stance phase. Further, in the slow leg at A0 and A2, this synergy represented activation of the TFL that was also active during the single limb support phase. Synergy #5 represented the activation of the BF and ST that occurred in the late swing phase. In the fast leg at A0, the BF and ST muscles were also active throughout the swing phase. Furthermore, in the fast leg at A0, a section-specific muscle synergy that was not sorted from Synergy #1 to #5 was extracted. This synergy represented the activation of the Gmed, BF, ST, MG, LG, and SOL during the initial double stance phase and the single limb support phase.

Regarding areas of the temporal activation patterns in the slow leg (Figure 4C), there was a significant main effect of section in Synergy #1 [Chi^2^ (df = 4) = 30.55, *P* < 0.05]. The area at A0 was significantly greater than that at A4, A6, and A8 (all *P* < 0.0051). The area at A2 was also significantly greater than that at A6 (*P* < 0.0051). In Synergy #2, a significant main effect of section was not observed [*F*_(2, 14)_ = 0.29, *P* > 0.05]. In Synergy #3, there was a significant main effect of section [*F*_(4, 44)_ = 3.02, *P* < 0.05], but the *post-hoc* tests did not show significant differences among sections. In Synergy #4, a significant main effect of section was shown [*F*_(1.83, 10.96)_ = 26.05, *P* < 0.05], and the area at A0 was significantly greater than that at the other sections (all *P* < 0.0051). In Synergy #5, there was also a significant main effect of section [Chi^2^ (df = 4) = 25.53, *P* < 0.05] and the area at A0 was significantly greater than that at A4, A6, and A8 (all *P* < 0.0051). For the fast leg (Figure 4F), there was no significant main effect of section in Synergy #1, #2, and #3 [Synergy #1: *F*_(4, 40)_ = 1.63; Synergy #2: *F*_(3,15)_ = 0.89; Synergy #3: *F*_(4, 44)_ = 1.10, all *P* > 0.05]. In Synergy #4, since only one participant recruited this muscle synergy at all sections, we did not perform a statistical analysis. In Synergy #5, a significant main effect of section was shown [Chi^2^ (df = 4) = 17.90, *P* < 0.05] and the area at A0 was significantly greater than that at A6 and A8 (both *P* < 0.0051).

### Extracted Muscle Synergies in Baseline and Post-adaptation Conditions

Figure 5 shows the average muscle weightings, average temporal activation patterns, and the areas under the curves of temporal activation patterns in each leg at each section in the baseline and post-adaptation conditions with the same convention as that of Figure 4. The principal muscles and main activation timings in each muscle synergy in baseline and post-adaptation conditions were almost the same as those in the adaptation condition (Tables 1, 2). However, some characteristic changes in the muscle synergies were observed among sections in the baseline and post-adaptation conditions. Synergy #2 was not extracted in both legs at P0. For Synergy #3 of the slow leg at P0, the activation level of the temporal activation patterns was high even in the early stance phase. In Synergy #4 of both legs, although the activation of the AL was mainly represented at all sections, the activation of the TFL and RF was also represented at P0. The muscles involved in this synergy were also active during the single limb support phase in the fast leg at P0. Further, in Synergy #5 at P0, additional activation was observed during the stance phase in the slow leg, and during the swing phase in the fast leg.

Regarding the areas of the temporal activation patterns in the slow leg (Figure 5C), there was a significant main effect of section in Synergy #1 and #5 [Synergy #1: *F*_(3, 21)_ = 11.22; Synergy #5: *F*_(3, 21)_ = 25.40, both *P* < 0.05]. The *post-hoc* tests showed that the area at P0 was significantly greater than that at the other sections (all *P* < 0.0085). In Synergy #2, there was no significant main effect of section [*F*_(2, 16)_ = 0.05, *P* > 0.05]. In Synergy #3, a main effect of section was significant [Synergy #3: Chi^2^ (df = 3) = 16.20, *P* < 0.05] and the area at P0 was significantly greater than that at BS and P4 (both *P* < 0.0085). In Synergy #4, a significant main effect of the section was observed [*F*_(3, 15)_ = 8.50, *P* < 0.05], and the area at P0 was significantly greater than that at P2 and P4 (both *P* < 0.0085). For the fast leg (Figure 5F), although a significant main effect of the section was shown in Synergy #1 [*F*_(3, 24)_ = 5.93, *P* < 0.05], significant differences among sections were not observed. In Synergy #2, since only three participants recruited this muscle synergy throughout all sections, statistical analyses were not conducted. In Synergy #3, there was a significant main effect of section [Chi^2^ (df = 3) = 8.70, *P* < 0.05], and the area at BS was significantly greater than that at P4 (*P* < 0.0085). In Synergy #4, a significant main effect was not observed [Chi^2^ (df = 3) = 7.00, *P* > 0.05]. In Synergy #5, there was a significant main effect of section [*F*_(3, 30)_ = 8.80, *P* < 0.05], and the area at P0 was significantly greater than that at the other sections (all *P* < 0.0085).

## Discussion

We studied changes in muscle synergies over time and tested which one of the four sub-hypotheses were valid in the adaptation and de-adaptation processes on the split-belt treadmill. The main findings were that the number of muscle synergies changed in the slow leg during split-belt walking and in both legs during tied-belt walking after split-belt walking. Moreover, one section-specific muscle synergy was extracted in the fast leg in the initial phase of split-belt walking. The areas of the temporal activation patterns in a few specific muscle synergies decreased during split-belt walking (Figures 4C,F). Meanwhile, the areas of the temporal activation patterns in a few specific muscle synergies increased temporally in the initial phase of tied-belt walking following split-belt walking and then decreased (Figures 5C,F). We discuss these changes in muscle synergies below.

### Changes in the Number of Extracted Muscle Synergies

The series of changes in the step symmetry identified in the present study (Figure 2) was almost consistent with those in previous studies that investigated the split-belt locomotor adaptation (Malone and Bastian, 2010; Bruijn et al., 2012; Yokoyama et al., 2018). We performed a muscle synergy analysis under the premise that split-belt locomotor adaptation had occurred. Overall, the number of muscle synergies extracted during split-belt and tied-belt walking (i.e., four or five muscle synergies) was similar to that identified in previous studies that had examined muscle synergies during walking (Clark et al., 2010; Dominici et al., 2011; Janshen et al., 2017). Thus far, some researchers have indicated the possibility that the number of muscle synergies does change depending on walking speeds (Yokoyama et al., 2016; Kibushi et al., 2018). Interestingly, in the present study, the number of muscle synergies changed at the constant walking speed (i.e., at 3.0 km/h in the slow leg in the adaptation condition or 3.0 km/h in both legs in the baseline and post-adaptation conditions). Specifically, the EMG data in the slow leg at A0 and A2, and in both legs at P0 were well accounted for by four muscle synergies temporally, not five. This result likely reflects that the independence of muscular control reduced immediately after exposure to the split-belt condition and tied-belt condition following the split-belt condition (Clark et al., 2010; Ting et al., 2015). On the other hand, each muscle synergy extracted during normal walking has been assumed to have particular functions (Neptune et al., 2009; Lacquaniti et al., 2012). In the present study, the muscle synergy that was not extracted at the section where the number of muscle synergies was four was Synergy #2. The muscle synergy mainly represented the activation of the TFL (Figures 4, 5), whose function is considered to be body stabilization (Rimini et al., 2017). The body stabilization might be complemented partially by Synergy #4 because the activation of the TFL was included in Synergy #4 immediately after changing walking conditions. Subsequently, since Synergy #2 might have become controllable independently as adaptation and de-adaptation progressed, the number of muscle synergies would have increased from four to five. Thus, the number of muscle synergies was shown to change according to the split-belt locomotor adaptation.

### Section-Specific Muscle Synergy

Although the change in the number of muscle synergies was not observed in the fast leg in the adaptation condition, a section-specific muscle synergy was included within the five muscle synergies extracted at A0 (Figures 4D,E). The muscle weightings of this muscle synergy consisted of the extensor muscles in the lower limb, which might work to maintain balance immediately after being imposed to walk at different belt speeds on the left and right sides. Subsequently, the section-specific muscle synergy might not be extracted after A2 because the balance was restored with adaptation to the disturbance caused by the unfamiliar walking environment. Thus, the muscle weightings were shown to change in the fast leg during split-belt walking. The appearance and disappearance of the extra muscle synergy are considered to reflect how the CNS deals with a new walking condition.

### Changes in the Temporal Activation Patterns of Extracted Muscle Synergies

In the adaptation condition, significant changes in the areas among sections were observed in three muscle synergies of the slow leg and one muscle synergy of the fast leg (Figures 4C,F). In a previous study, it was reported that the temporal activation patterns changed abruptly without changes in muscle weightings in several muscle synergies just after a robot-driven perturbation was given to the lower limb during walking (Severini et al., 2020). The abrupt changes in the temporal activation patterns are considered to be a reactive response to the perturbation *via* the feedback mechanism. Thus, the great areas in the initial phase of the adaptation condition might also reflect a reactive response to novel constraints with a split-belt treadmill at the muscle synergy level (Hoogkamer, 2017; Severini et al., 2020). On the other hand, since we analyzed muscle synergies within the same belt condition (i.e., within the split-belt condition or tied-belt condition), biomechanical task constraints that would affect muscle synergies are believed to be constant. Nevertheless, the areas of a few specific muscle synergies decreased during split-belt walking (Figures 4C,F), which would result from mainly a decrease in activation levels in the temporal activation patterns. For Synergy #4 in the slow leg, a decrease in the area would result from the disappearance of an additional activation in the stance phase. The manners of these decreases in the areas, in a few specific muscle synergies, appear to be gradual. Thus, this result likely reflects that temporal activation patterns were adjusted *via* the feedforward mechanism (Severini et al., 2020). These adaptive changes in the temporal activation patterns have also been observed by repeating an imposed walking task in another previous study (Martino et al., 2015). The feedforward control is characterized by an aftereffect, occurring when a perturbation was removed (Torres-Oviedo et al., 2011). In the present study, the greater areas at P0 than at BS have been observed in several muscle synergies (Figures 5C,F), suggesting that aftereffect occurred at the muscle synergy level. The aftereffect was then washed out and the areas returned to the baseline level. The series of changes in the areas imply that adaptation and de-adaptation occurred in the temporal activation patterns of a few specific muscle synergies in association with the split-belt locomotor adaptation.

Regarding the feedforward control, as mentioned in the previous paragraph, the supraspinal structures have been suggested to be involved (Choi et al., 2009). Therefore, the changes in the temporal activation patterns observed in the present study might reflect that the involvement of supraspinal structures changed in the split-belt locomotor adaptation. In particular, since cortical activation has been reported to be related to the activation of muscle synergies during walking (Yokoyama et al., 2019), it is considered that the involvement of the cortex changed. The changes in the cortical involvement associated with the split-belt locomotor adaptation have also been indicated in recent studies using the EMG-EMG coherence analysis, albeit at the individual muscle level (Sato and Choi, 2019; Oshima et al., 2021). Additionally, afferent signals from the lower limb have been considered to be one of the factors that influence the activation of muscle synergies in animal studies (Cheung et al., 2005; Bizzi and Cheung, 2013). The amount of various somatosensory information would change during split-belt walking (Hoogkamer, 2017). Ogawa et al. (2014) showed that the magnitude of the GRF associated with the load-related sensory information significantly changed during split-belt walking. Therefore, changes in both supraspinal origin and somatosensory information might be related to changes in the temporal activation patterns in a few specific muscle synergies.

It should be noted that the changes in the areas were more prominent in the slow leg than in the fast leg (Figure 4). The results would indicate that the CNS tried to adjust the slow leg rather than the fast leg. This idea appears to be consistent with the contention of a previous study that the CNS might give importance to a slow leg during split-belt walking (Vasudevan and Bastian, 2010). Thus, the significant changes in the areas would be observed in more muscle synergies of the slow leg compared with the fast leg as an aftereffect when the belts returned to the same speed (Figure 5).

### Limitation

The limitation of the present study is that the participants were limited to young males. It is thus unclear whether the observed changes in muscle synergies occur in other age groups. Further research will be required to answer this question, likely contributing toward understanding the differences in the ability to adapt walking patterns between younger and older subjects (Bruijn et al., 2012; Sato and Choi, 2021). As a methodological concern, it should be kept in mind that the low-pass cut-off frequency influences the number of extracted muscle synergies (Turpin et al., 2021).

## Conclusion

Our results showed changes in the number of muscle synergies, appearance and disappearance of extra muscle synergy, and modulation of the temporal activation patterns of a few specific muscle synergies in the adaptation and de-adaptation processes on the split-belt treadmill. These results support hypothesis 4 and suggest that adaptation and de-adaptation have occurred at the muscle synergy level. The understanding of neural control strategies underlying the split-belt locomotor adaptation is advanced by our findings, based on the muscle synergy concept.

## Data Availability Statement

The raw data supporting the conclusions of this article will be made available by the authors, without undue reservation.

## Ethics Statement

The studies involving human participants were reviewed and approved by the Doshisha University Research Ethics Review Committee regarding Human Subject Research. The participants provided their written informed consent to participate in this study.

## Author Contributions

AO and KK contributed to the conception, design of the study, interpreted the experimental results, edited, and revised the manuscript. AO performed the experiments, analyzed the data, wrote the draft of the manuscript, and prepared the figures. All authors contributed to the article and approved the submitted version.

## Conflict of Interest

The authors declare that the research was conducted in the absence of any commercial or financial relationships that could be construed as a potential conflict of interest.

## Publisher’s Note

All claims expressed in this article are solely those of the authors and do not necessarily represent those of their affiliated organizations, or those of the publisher, the editors and the reviewers. Any product that may be evaluated in this article, or claim that may be made by its manufacturer, is not guaranteed or endorsed by the publisher.

## Footnotes

1
http://www.seniam.org
